# On the Coexistence of the Carbene⋯H-D Hydrogen Bond and Other Accompanying Interactions in Forty Dimers of N-Heterocyclic-Carbenes (I, IMe_2_, I^*i*^Pr_2_, I^*t*^Bu_2_, IMes_2_, IDipp_2_, IAd_2_; I = imidazol-2-ylidene) and Some Fundamental Proton Donors (HF, HCN, H_2_O, MeOH, NH_3_)

**DOI:** 10.3390/molecules27175712

**Published:** 2022-09-05

**Authors:** Mirosław Jabłoński

**Affiliations:** Faculty of Chemistry, Nicolaus Copernicus University in Toruń, ul. Gagarina 7, 87-100 Toruń, Poland; teojab@chem.umk.pl; Tel.: +48-056-611-4695

**Keywords:** carbene, N-heterocyclic carbene, NHC, imidazol-2-ylidene, hydrogen bond, intermolecular interaction, secondary interaction, organometallic chemistry, DFT

## Abstract

The subject of research is forty dimers formed by imidazol-2-ylidene (I) or its derivative (IR${}_{2}$) obtained by replacing the hydrogen atoms in both N-H bonds with larger important and popular substituents of increasing complexity (methyl = Me, *iso*-propyl = ${}^{i}$Pr, *tert*-butyl = ${}^{t}$Bu, phenyl = Ph, mesityl = Mes, 2,6-diisopropylphenyl = Dipp, 1-adamantyl = Ad) and fundamental proton donor (HD) molecules (HF, HCN, H${}_{2}$O, MeOH, NH${}_{3}$). While the main goal is to characterize the generally dominant C⋯H-D hydrogen bond engaging a carbene carbon atom, an equally important issue is the often omitted analysis of the role of accompanying secondary interactions. Despite the often completely different binding possibilities of the considered carbenes, and especially HD molecules, several general trends are found. Namely, for a given carbene, the dissociation energy values of the IR${}_{2}\cdots$HD dimers increase in the following order: NH${}_{3}$< H${}_{2}$O < HCN ≤ MeOH ≪ HF. Importantly, it is found that, for a given HD molecule, IDipp${}_{2}$ forms the strongest dimers. This is attributed to the multiplicity of various interactions accompanying the dominant C⋯H-D hydrogen bond. It is shown that substitution of hydrogen atoms in both N-H bonds of the imidazol-2-ylidene molecule by the investigated groups leads to stronger dimers with HF, HCN, H${}_{2}$O or MeOH. The presented results should contribute to increasing the knowledge about the carbene chemistry and the role of intermolecular interactions, including secondary ones.

## 1. Introduction

In carbenes [1,2,3,4,5,6,7,8,9,10,11,12,13,14,15,16,17,18,19,20,21,22,23,24,25,26,27,28,29,30,31,32,33,34,35,36,37,38,39,40,41,42,43,44,45,46,47,48,49,50,51,52,53,54,55,56,57,58,59,60,61,62,63,64,65,66,67,68,69,70,71,72,73,74,75,76,77,78,79,80], the carbon atom is $sp^{2}$-hybridized. However, unlike the vast majority of organic compounds, in carbenes, it is merely divalent, consequently forming only one (R=C) or at most two (R${}_{1}$R${}_{2}$C) covalent bonds. Thus, two valence electrons remain available, giving the possibility of two spin states, singlet or triplet [5,6,8,70]. In the spin singlet state, both electrons are paired to form a lone electron pair. Additionally, the carbene carbon atom has an unfilled *p*-orbital perpendicular to the $sp^{2}$ hybrids. While the triplet state is also possible in some carbenes, a number of factors are known to increase singlet stability. The close presence of a strongly electronegative atom, such as, e.g., N, is important here, which stabilizes the singlet state through both the inductive σ-electron withdrawing effect and the π-electron charge donation from the lone electron pair of this atom to the vacant *p*-orbital of the carbene carbon atom [5,6,8,18,70]. In the case of cyclic carbenes, another factor stabilizing the singlet state is the π-electron delocalization within the entire ring, which may be related to its aromaticity, as is the case, for example, in the imidazol-2-ylidene molecule [38]. Another factor that stabilizes the singlet state of a carbene is a low value of the R${}_{1}$–C–R${}_{2}$ angle [3,4,5]. Naturally, this condition is obviously present in cyclic carbenes. While all of the conditions mentioned so far exist in imidazol-2-ylidene, they need not necessarily occur simultaneously in other N-heterocyclic carbenes (NHCs) [61]. In their case, an important stabilizing factor is the size of the substituents attached to both nitrogen atoms, as larger substituents prevent carbene dimerization [1].

The presence of a lone electron pair on the carbene carbon atom makes carbenes good Lewis bases, showing strongly nucleophilic properties. Carbenes are mainly known for their association with transition metal atoms (see, e.g., refs. [11,30,31,36,55,63]), making them extremely useful compounds in organic, organometallic and materials chemistry as well as homogeneous catalysis [21,30,33,57,61,62,69]. In addition to bonds with metals, however, carbenes quite willingly also form other intermolecular connections, such as hydrogen bonds [9,10,16,19,38,51,56,58], lithium bonds [39,45,46,62,69], beryllium bonds [17,53,54,62,69,73,79], magnesium bonds [14,22,40,41,62,69,73,79], triel bonds [13,20,23,27,69], tetrel bonds [42,65,66,69], pnictogen bonds [47,48,50,69], chalcogen bonds [52,69], halogen bonds [12,15,43,59,69] (in particular to iodine [12,15]), and aerogen bonds [67]. Moreover, in addition to these possibilities resulting from the presence of a lone electron pair, the presence of an empty *p* orbital in singlet carbenes also gives them electrophilic properties that seem to be much less studied [7,24,29,68,71,74,75].

Considering the fundamental role of hydrogen bonds [81,82,83,84,85,86,87,88,89,90,91,92,93,94,95,96], it is somewhat surprising that hydrogen bonds involving carbenes are studied only very sporadically [9,10,16,19,38,51,56,58]. Obviously, due to considerable methodological limitations, the first theoretical reports (in 1983 by Pople et al. [9] and then in 1986 by Pople [10]) concerned a simple H${}_{2}$C⋯HF dimer. Then, in 1996, Alkorta and Elguero investigated dimers between H${}_{2}$C or F${}_{2}$C and a few simple proton donors [19]. More recently, Jabłoński and Palusiak studied hydrogen bonds between carbenes CF${}_{2}$, CCl${}_{2}$ and imidazol-2-ylidene and such proton donors as H${}_{2}$O and HCF${}_{3}$ [38]. In a wonderful extensive work on the application of theoretical methods in the study of carbene chemistry, Gerbig and Ley [56] also mentioned the imidazol-2-ylidene⋯HCF${}_{3}$ dimer in which there is C⋯H-C hydrogen bonding and the accompanying N-H⋯F (both described earlier in ref. [38]). Samanta et al. [58] showed the possibility of the formation of either C⋯H-N or C⋯H-O hydrogen bonds between the simple heterocyclic derivative of 1,3-di(methyl)imidazol-2-ylidene (IMe${}_{2}$) and MeNH${}_{2}$ or, in particular, MeOH, respectively. In the latter case, the NHC acts as an esterification catalyst activating the alcohol molecule. On the other hand, from the experimental point of view, hydrogen bonding with carbene was first announced by Arduengo et al. [16] in 1995 with the report essentially relating to the C–H–C bridge in a bis(carbene)–proton complex formed by 1,3-di(2,4,6-trimethylphenyl)-imidazol-2-ylidene (IMes${}_{2}$). Much later, in 2011, it was shown that IMes${}_{2}$ and 1,3-di(2,6-diisopropylphenyl)imidazolidin-2-ylidene (SI${}^{i}$Pr${}_{2}$) can form a C⋯H-O type hydrogen bond to 1-hydroxy-2,2,6,6-tetramethyl-piperidine (TEMPO-H) [51]. In addition to crystallographic and NMR studies, results of theoretical calculations were also reported.

**Figure 1:**
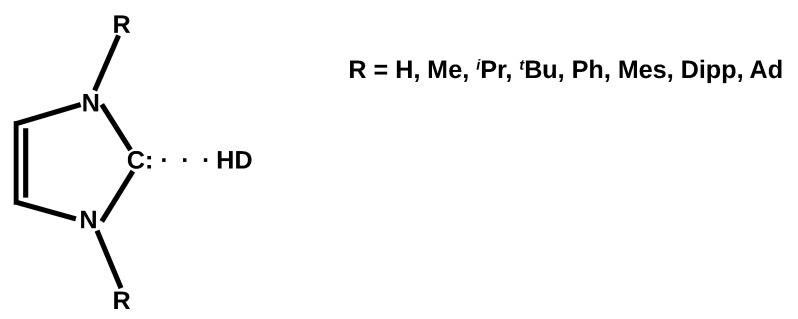
General scheme of the IR${}_{2}\cdots$HD dimers (R = H, Me, ${}^{i}$Pr,${}^{t}$Bu, Ph, Mes, Dipp, Ad). The colon on the carbene carbon atom represents a lone electron pair.

As shown above, the theoretical studies for hydrogen bonding involving carbenes have generally been down to very small carbenes, at most imidazol-2-ylidene (I) or 1,3-di(methyl)imidazol-2-ylidene (IMe${}_{2}$). However, there are no theoretical studies in which the imidazol-2-ylidene derivative would contain even larger substituents on both nitrogen atoms. Moreover, the influence of the presence of these substituents on the possible interactions accompanying the leading C⋯H-D hydrogen bond has not been investigated yet. This article aims to fill this gap. Namely, this article examines the hydrogen bonds between imidazol-2-ylidene (I) and its seven popular derivatives containing gradually more bulky substituents (methyl = Me, *iso*-propyl = ${}^{i}$Pr, *tert*-butyl = ${}^{t}$Bu, phenyl = Ph, mesityl = Mes, 2,6-diisopropylphenyl = Dipp, 1-adamantyl = Ad) on both nitrogen atoms and five fundamental proton donor molecules (HF, HCN, H${}_{2}$O, MeOH, NH${}_{3}$). The possible combinations form forty dimers, which can be briefly designated as IR${}_{2}\cdots$HD, where R is one of the substituents mentioned previously. The general scheme of the dimers in question is shown in Figure 1.

It should be emphasized that the C⋯H-D hydrogen bonds studied here engage the carbene species in their singlet spin states and thus a lone electron pair on the carbene carbon atom (Figure 1) and are therefore considerably distant from hydrogen bonds involving radicals [97,98]. In addition to the description of C⋯HD hydrogen bonds, an equally important goal is to analyze the possibility of the emergence of various types of accompanying interactions and their impact on the structure of the obtained dimers. This concerns the important issue of the coexistence of various types of interactions in molecular systems. Recently, the author of the present paper has shown [80] that various types of secondary interactions have a significant effect on the mutual orientation of the ZnX${}_{2}$ molecular plane relative to the imidazol-2-ylidene ring in various types of IR${}_{2}$ carbenes. This result is important because the possible torsion of the planes is usually attributed to the steric effects resulting from the presence of bulky R substituents.

## 2. Theoretical Methods

Geometries of all the systems were fully optimized on the ωB97X-D/6-311++G(d,p) level of theory, i.e., using the ωB97X-D range-separated gradient- and dispersion-corrected hybrid exchange-correlation functional [99] of density functional theory [100,101,102] and the 6-311++G(d,p) basis set being of the triple-zeta type and containing both polarization and diffuse functions on all atoms [103,104,105,106,107]. It is worth noting that ωB97X-D was one of the best functionals out of 200 tested [108]. There were no imaginary frequencies showing that equilibrium structures were obtained each time. Both the geometry optimization and the frequency analysis were performed using the Gaussian 16 (Revision C.01) program [109].

Values of the electron density at the bond critical points (bcp) [110,111,112] of the C⋯H-D hydrogen bonds and other accompanying interactions were computed using the AIMAll program [113]. Indeed, the C⋯H-D hydrogen bond should also be investigated through quantum chemical topology, which, over the years, was shown to be an efficient theoretical approach [114,115].

The dissociation energy was calculated as the difference between the total energy of a dimer and the sum of total energies of isolated subsystems in their own fully optimized structures. Total energies were corrected for the zero-point vibrational energies (ZPVE). Dissociation energies are given as positive values. In order to determine the binding energy (in fact, the interaction energy [116]) of the individual interaction of interest, the formula suggested recently by Emamian et al. [116] was used:

(1) $$E_{b}\left[\mathrm{kcal}/\mathrm{mol}\right]=-223.08\cdot \rho_{\mathrm{bcp}}\left[a.u.\right]+0.7423$$

This formula is based on the electron density value determined at the bond critical point of this interaction, which is the parameter that, as has been shown [116], is best correlated with binding energy among many wave function-based HB descriptors. As a result, Emamian et al. proposed using this equation for a quick estimate of the energy of hydrogen-bond-forming networks. It is worth emphasizing that the values of the dissociation energies and the (sum) of the binding energies determined by Equation (1) are not comparable, since the former is a global quantity relating to the entire dimer, while the latter is a local parameter relating to an individual interaction. Besides, the dissociation energy takes into account deformation energies and ZPVEs.

## 3. Results and Discussion

### 3.1. Hydrogen Bonds to Carbenes Observed in the Solid State

**Figure 2:**
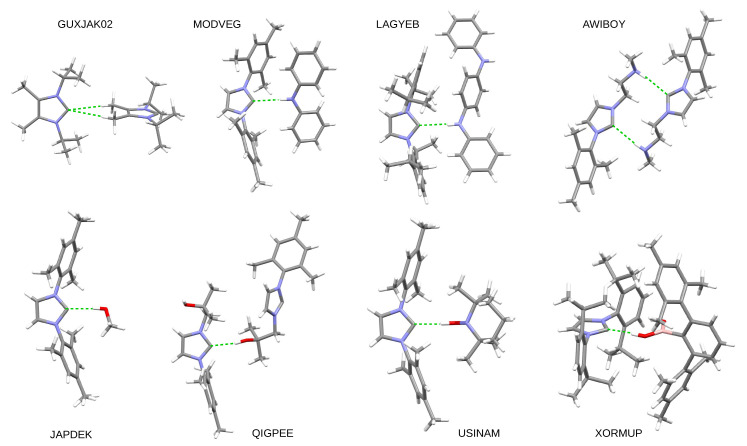
Examples of the occurrence of hydrogen bonds (marked with a dashed green line) involving a carbene carbon atom in crystallographic structures. The atoms are labeled as follows: carbon—gray, hydrogen—white, nitrogen—blue, oxygen—red, and boron—pink.

It should be noted that despite the high reactivity of carbene compounds, hydrogen bonds with the participation of the carbene carbon atom as a proton acceptor quite often occur in the crystals. Of course, due to the abundance of C-H bonds, the most common hydrogen bond of this type is C⋯H-C, but C⋯H-N or C⋯H-O can also be found in the Cambridge Structural Database [117]. Some more interesting examples are shown in Figure 2.

There are two C⋯H-C type hydrogen bonds in the crystal form of 1,3-diisopropyl-4,5-dimethylimidazol-2-ylidene (GUXJAK02) [118]. Both the dimer between 1,3-dimesityl-imidazol-2-ylidene and diphenylamine (MODVEG) [119] and between 1,3-bis[2,6- diisopropylphenyl]imidazol-2-ylidene and N${}^{1}$,N${}^{4}$-diphenylbenzene-1,4-diamine (LAGYEB) [120] have one C⋯H-N hydrogen bond, while the dimer of N-methyl-2-(3-phenyl-2,3-dihydro-1H-imidazol-1-yl)ethanamine (AWIBOY) [121] has two. 1,3-dimesitylimidazol-2-ylidene in methanol (JAPDEK) [122], on the other hand, is the simplest example of the occurrence of a C⋯H-O type hydrogen bond and was reported as the first X-ray structure of a carbene–alkohol hydrogen-bonded complex. The -OH group donors are somewhat more complex in QIGPEE (i.e., the dimer of 1-(2-hydroxy-2-methylpropyl)-3-(2,4,6-trimethylphenyl)-imidazol-2-ylidene) [123], USINAM (i.e., the dimer between 1,3-dimesitylimidazol-2-ylidene and 2,2,6,6-tetramethylpiperidin-1-ol) [51], and XORMUP (the dimer between 1,3-bis[2,6-bis(propan-2-yl)phenyl]-imidazol-2-ylidene and (2,4,6,2${}^{\prime \prime }$,4${}^{\prime \prime }$,6${}^{\prime \prime }$-hexamethyl[1,1${}^{\prime }$:3${}^{\prime }$,1${}^{\prime \prime }$-terphenyl]-2${}^{\prime }$-yl)boronic acid) [124]. Interestingly, in the last system, there is the -B(OH)${}_{2}$ group, of which, however, only one -O-H bond is involved in the formation of the C⋯H-O bridge with the carbene molecule.

The examples presented show clearly that the carbene carbon can actually engage in hydrogen bonding quite easily by acting as a proton acceptor. It can also be seen that these carbenes have large substituents (most often in the form of mesityl groups) in the 1 and 3 positions of the imidazol-2-ylidene parent molecule. Therefore, the study of these types of systems using theoretical methods is also very important [80]. However, the presence of bulky substituents significantly increases the size of the systems and thus significantly increases the computational cost of theoretical research. Moreover, their presence increases the probability of the occurrence of interactions other than C⋯H-D, which additionally complicates the research. Probably for these reasons the amount of theoretical studies on this type of systems is small. As stated in the Introduction, the purpose of this article is to fill this gap to some extent. The investigated systems will be described in the next subsection.

### 3.2. Investigated Systems

The subject of the research in this article is the hydrogen bonds between imidazol-2-ylidene (I) and its seven derivatives having a pair of R substituents (methyl = Me, *iso*-propyl = ${}^{i}$Pr, *tert*-butyl = ${}^{t}$Bu, phenyl = Ph, mesityl = Mes, 2,6-diisopropylphenyl = Dipp, 1-adamantyl = Ad) attached to both nitrogens and five popular and important H-D proton donors (HF, HCN, H${}_{2}$O, MeOH, NH${}_{3}$). Dimers (complexes) having this bond will be denoted as IR${}_{2}\cdots$HD for simplicity. Apart from the description of C⋯H-D hydrogen bonds in these systems, an important topic will be the possible presence of other accompanying bonds as they can sometimes significantly influence the structure of a complex [80]. Values of some selected parameters characterizing the C⋯H-D hydrogen bond or the proton-donor molecule in the IR${}_{2}\cdots$HD dimers are listed in Table 1. Because imidazol-2-ylidene is somewhat different in terms of its binding capacity, the imidazol-2-ylidene complexes will be discussed first.

### 3.3. Imidazol-2-ylidene Complexes

**Figure 3:**
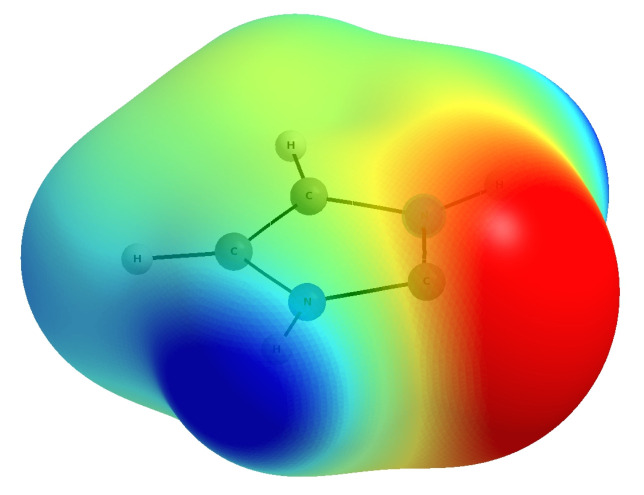
Map of the electrostatic potential (in a.u.) of imidazol-2-ylidene: −0.06, red; −0.03, yellow; 0.00, green; 0.03, cyan; 0.06, blue.

Compared to all other molecules, imidazol-2-ylidene is distinguished by the presence of highly polar N-H bonds (Figure 3), which are good proton donors and therefore can eagerly form hydrogen bonds as long as there is an atom with good proton-acceptor properties, such as O, N or a halogen atom, in their presence.

**Figure 4:**
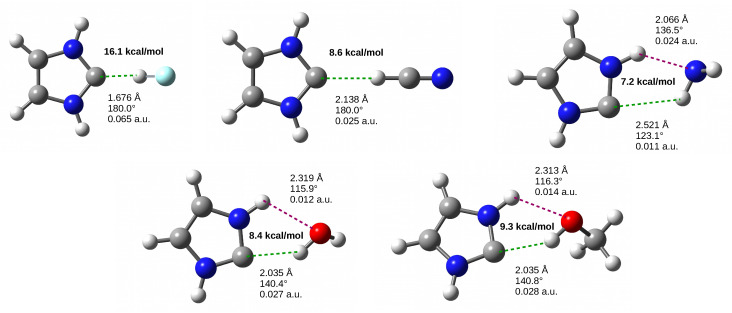
Imidazol-2-ylidene dimers with HF, HCN, NH${}_{3}$, H${}_{2}$O and MeOH. The values of the dissociation energy are given in bold, while the three values next to the interaction shown refer to the length of the hydrogen bond, its angle and the value of the electron density at the bond critical point.

The stable dimers between imidazol-2-ylidene and HF, HCN, H${}_{2}$O, MeOH or NH${}_{3}$ are shown in Figure 4.

In the case of HF and HCN, the obtained hydrogen bonds are linear, the former of which is very strong, which is expressed by a large value of the determined dissociation (16.1 kcal/mol) and binding (−13.7 kcal/mol) energies, a short distance C⋯H (1.676 Å) and a fairly high value of the electron density determined at the critical point ($\rho_{\mathrm{bcp}}$) of this bond (0.065 a.u.). In addition, the formation of the C⋯H-F hydrogen bond leads to a significant elongation of the H-F bond (+0.061 Å) and a very large red-shift of its stretching vibration frequency (−1317 cm${}^{-1}$). On the other hand, the interaction in the I⋯HCN dimer is almost half as weak (8.6 kcal/mol), which is also related to the much longer C⋯H distance (2.138 Å) and the much lower $\rho_{\mathrm{BCP}}$ value (0.025 a.u.). The elongation of the C-H bond in HCN and the red-shift of its stretching vibration frequency are only +0.029 Å and −397 cm${}^{-1}$, respectively.

As can be seen in Figure 4, unlike I⋯HF and I⋯HCN, in the remaining systems, i.e., I⋯NH${}_{3}$, I⋯H${}_{2}$O and I⋯MeOH, the C⋯H-D hydrogen bond is not linear due to the presence of another hydrogen bond. The presence of this accompanying hydrogen bond results not only from the presence of a strongly polar N-H bond in the imidazol-2-ylidene molecule, but above all from the presence of the D atom having an easily accessible lone electron pair. This possibility leads to the formation of the N-H⋯O hydrogen bond in the dimers I⋯H${}_{2}$O and I⋯MeOH and N-H⋯N in the I⋯NH${}_{3}$ dimer. Importantly, this situation, i.e., the simultaneous presence of two strong hydrogen bonds, means that the determined values of the dissociation energy do not represent strengths of the C⋯HD hydrogen bonds only, because, as representing intermolecular interactions globally, they should rather be assigned to both the hydrogen bonds, i.e., C⋯H-D and the accompanying one. Moreover, it is not easy to extract energy values for individual bonds. Nevertheless, as already mentioned in the Theoretical Methods section, Emamian et al. [116] have recently shown that among many wave function-based HB descriptors (including those based on QTAIM), the electron density determined at the bond critical point of HB best correlates with the binding (in fact, interaction) energy of this HB, giving Equation (1). As a result, they proposed using this equation for a quick estimate of the energy of hydrogen bonds forming networks [116]. Using this proposal, in the case of the I⋯NH${}_{3}$ dimer, the value is −4.6 kcal/mol for the N-H⋯N hydrogen bond and only −1.7 kcal/mol for C⋯H-N. Thus, the N-H⋯N bond is definitely stronger than N-H⋯C, which is also expressed in a definitely shorter distance H⋯N (2.066 Å) than H⋯C (2.521 Å). However, in the case of I⋯H${}_{2}$O and I⋯MeOH dimers, the O-H⋯C bond is definitely stronger (−5.3 and −5.5 kcal/mol, respectively) than the accompanying N-H⋯O bond (−1.9 and −2.4 kcal/mol, respectively). This is also reflected in shorter C⋯H distance (2.035 Å) than H⋯O (2.319 and 2.313 Å, respectively).

The dimers I⋯NH${}_{3}$, I⋯H${}_{2}$O and I⋯MeOH are good simple examples clearly showing that imidazol-2-ylidene willingly forms accompanying hydrogen bonds by the strongly polar N-H bond. The formation of such bonds is particularly easy when the D atom has lone electron pairs readily available, as is the case, for example, of N and O atoms. The formation of these bonds is prevented after replacing the H atoms in both N-H bonds with non-polar R substituents. However, in addition to the frequently mentioned steric effects, this type of modification of the imidazol-2-ylidene molecule may lead to other accompanying secondary interactions, which is discussed in the other subsections.

### 3.4. The IR${}_{2}\cdots$HD Dimers

#### 3.4.1. The IR${}_{2}\cdots$HF and IR${}_{2}\cdots$HCN Dimers

**Figure 5:**
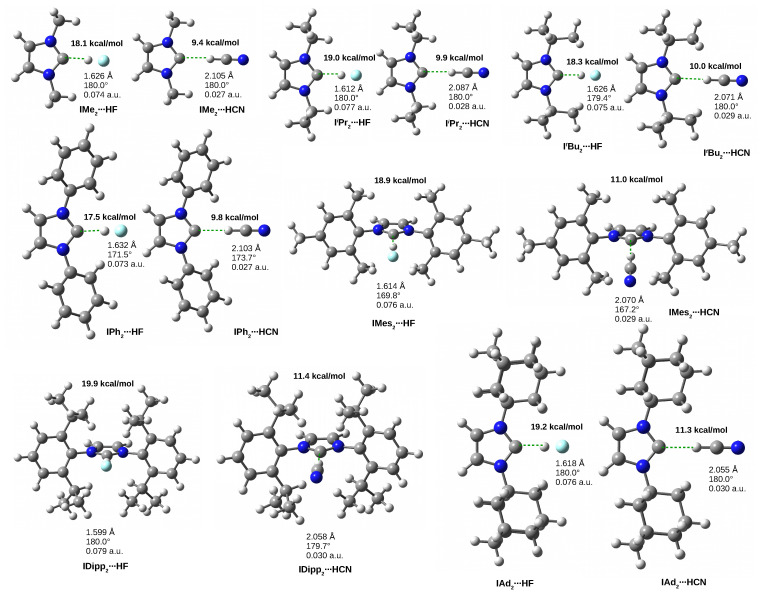
The IR${}_{2}\cdots$HF and IR${}_{2}\cdots$HCN (R = Me, ${}^{i}$Pr, ${}^{t}$Bu, Ph, Mes, Dipp, Ad) dimers. The values of the dissociation energy are given in bold, while the three values refer to the length of the hydrogen bond, the C-H-D angle and the value of the electron density at the bond critical point of the C⋯H-D hydrogen bond.

Due to the fairly large similarity in the values of the C-H-D angle, i.e., the local geometry of the C⋯H-D hydrogen bond, in the HF and HCN dimers, it is convenient to consider these dimers together. The fully optimized structures of the dimers IR${}_{2}\cdots$HF and IR${}_{2}\cdots$HCN are shown in Figure 5 along with the obtained values of dissociation energy, distance C⋯H, angle C-H-D and value of the electron density at the bond critical point of the C⋯H hydrogen bond.

First, let us note that, as with the I⋯HF and I⋯HCN dimers (Figure 4), the HF dimers are much stronger than their HCN counterparts. The dissociation energies are in the 17.5–19.9 kcal/mol range while the HCN dimer values are only 9.4–11.4 kcal/mol. In the former case, the strongest complex is IDipp${}_{2}\cdots$HF, and the weakest is IPh${}_{2}\cdots$HF, while in the latter, the strongest complex is also the one involving IDipp${}_{2}$, but the weakest dimer is formed by IMe${}_{2}$ instead. Much greater strength of hydrogen bonds C⋯H in IR${}_{2}\cdots$HF dimers than IR${}_{2}\cdots$HCN is also visible in much smaller distances H⋯C (1.599–1.632 Å vs 2.055–2.105 Å), much higher values of $\rho_{\mathrm{bcp}}$ (0.073–0.079 a.u. vs 0.027–0.030 a.u.), much greater extensions of the H-D bond (0.075–0.088 Å vs 0.031–0.040 Å) and much higher values of the $\nu_{\mathrm{HD}}$ stretching vibration frequency red-shift (from −1591 up to −1814 cm${}^{-1}$ vs. from −462 up to −548 cm${}^{-1}$). It is worth noting that although the dimers involving Ad (i.e., IAd${}_{2}\cdots$HF and IAd${}_{2}\cdots$HCN) are not the strongest (although the difference compared to their counterparts involving Dipp is small, especially in the case of the HCN dimer), the effect of extending the proton-donor bond and red-shift values are in these systems greatest. Therefore, it can be concluded that together with IDipp${}_{2}$, IAd${}_{2}$ also forms strong C⋯H hydrogen bonds, which have the greatest impact on the characteristics of HF and HCN proton-donor molecules. It is also worth noting that for both HF and HCN, the dissociation energies obtained for the dimers shown in Figure 5 are clearly greater than the energies obtained for I⋯HF and I⋯HCN (16.1 and 8.6 kcal/mol, respectively, as shown in Table 1 and Figure 4), and thus the substitution of hydrogen atoms in both N-H bonds in imidazol-2-ylidene even by methyl groups increases the strength of the dimer. This can most likely be explained by the weak inductive effect of these groups, which increases the charge on lone electron pairs.

It may be asked why the systems with IDipp${}_{2}$ and IAd${}_{2}$ are characterized by the highest values of dissociation energies (of course also the shortest H⋯C distances and the highest $\rho_{\mathrm{bcp}}$, $\Delta d_{\mathrm{HD}}$ and red-shift values). Very often, the increase in dimer strength is due to the presence of additional competing interactions, the presence of which is often suggested by the significant non-linearity of the dominant hydrogen bond, as was the case with the dimers I⋯H${}_{2}$O, I⋯MeOH and I⋯NH${}_{3}$ shown in Figure 4 and discussed earlier. However, the reference to the C-H-D angle value can be very deceptive because these values for both IDipp${}_{2}$ and IAd${}_{2}$ are exactly 180${}^{\circ }$, so the C⋯H-D hydrogen bonds in the dimers of these carbenes with HF and HCN are linear. What is more, interestingly, carbenes IPh${}_{2}$, and especially IMes${}_{2}$, are characterized by a distinct nonlinearity of C⋯H-D hydrogen bonds. In these cases, the non-linearity may indeed result from the presence of other competing interactions which affect the alignment of the proton-donor molecule and thus also the geometry of the C⋯H-D hydrogen bond.

**Figure 6:**
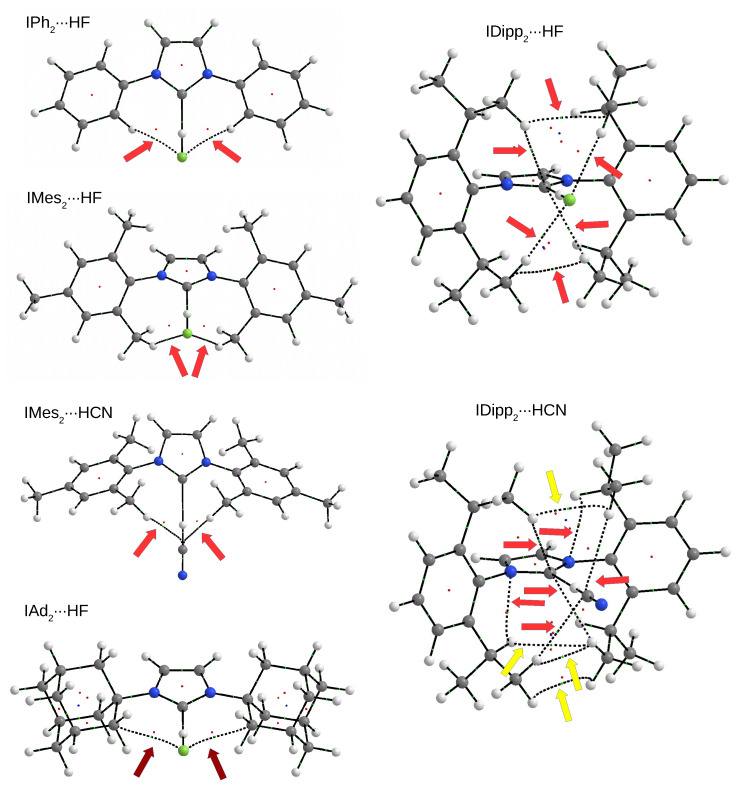
Molecular graphs of selected IR${}_{2}\cdots$HF and IR${}_{2}\cdots$HCN dimers. Arrows show the presence of bond paths for some accompanying interatomic interactions (hydrogen bonds—red, tetrel bonds—brown, and C-H⋯H-C contacts—yellow). Large balls represent atoms (hydrogen—white, carbon—gray, nitrogen—blue, and fluorine—light green) and small balls represent critical points (bond critical points—light green, ring critical points—red, and cage critical points—blue).

In order to search for such competitive interactions, the determination of a molecular graph defined by QTAIM [110,111,112] may be a particularly helpful tool. Figure 6 presents molecular graphs of several selected IR${}_{2}\cdots$HF and IR${}_{2}\cdots$HCN dimers.

As can be clearly seen, in each of the examples, the molecular graph shows the presence of accompanying interatomic interactions, which are indicated by color-coded arrows. IPh${}_{2}\cdots$HF is a simple example in which the molecular graph suggests the presence of two accompanying C-H⋯F type hydrogen bonds. A similar situation occurs in the slightly larger IMes${}_{2}\cdots$HF, where the proton-donating C-H bond is derived from one of the methyl groups in the mesityl group. Both these bonds should be very weak, which is suggested by low electron density values (ca. 0.008 a.u.). Using Equation (1) gives the value of −1.0 kcal/mol. In contrast, in the IMes${}_{2}\cdots$HCN dimer, a similar pair of bond paths indicates the presence of CH⋯C-type hydrogen bonds, which should be even weaker ($\rho_{\mathrm{bcp}}$ amounts to ca. 0.0034 a.u. only, and therefore, the bonding effect is negligible) than C-H⋯F. The IAd${}_{2}\cdots$HF dimer is a very simple example of a system featuring a C⋯F tetrel bonding pair. This time the binding energy value is significant (ca. −1.4 kcal/mol), suggesting that the pair of these interactions may contribute significantly to the overall binding effect of the dimer.

The presence of 2,6-diisopropylphenyl (Dipp) substituent allows a particularly large number of accompanying interactions [80]. Apart from pairs of hydrogen bonds of the type C⋯H-F (or C⋯H-C in IDipp${}_{2}\cdots$HCN), both these dimers, i.e., IDipp${}_{2}\cdots$HF and IDipp${}_{2}\cdots$HCN, experience the presence of bond paths between the hydrogen atom of a C-H bond and the carbene carbon atom. Therefore, this result suggests that apart from the dominant C⋯H-D hydrogen bond (D = F or C), the carbene carbon atom engages in two additional C⋯H-C hydrogen bonds, which, however, should be very weak (ca. −0.9 and −1.1 kcal/mol in IDipp${}_{2}\cdots$HF and IDipp${}_{2}\cdots$HCN, respectively). In the case of IDipp${}_{2}\cdots$HCN, the molecular graph also suggests the presence of a pair of *intra*molecular hydrogen bonds of the C-H⋯N type, as nitrogen atoms belong to the imidazol-2-ylidene ring. Since the values of $\rho_{\mathrm{bcp}}$ are significant (ca. 0.016 a.u.), the use of Equation (1) yields the value of ca. −2.8 kcal/mol. Apart from the aforementioned accompanying hydrogen bonds, the molecular graphs of dimers IDipp${}_{2}\cdots$HF and IDipp${}_{2}\cdots$HCN also show the presence of numerous C-H⋯H-C interactions, which, however, seems to be a fairly common feature in the case of crowded systems with many C-H bonds [80,125,126,127].

As shown (Figure 6), molecular graphs suggest the presence of many different interactions, including intermolecular ones. Their role in the overall binding of the system and their influence on the overall structure is not easy to quantify, although there are many descriptors for individual, i.e., local, interactions [116]. One such descriptor is the electron density value determined at the bond critical point of a given interaction, which, as already mentioned, best correlates with the binding energy [116]. In the case of IR${}_{2}\cdots$HF and IR${}_{2}\cdots$HCN dimers, the estimates based on Equation (1) show that these interactions should be much weaker than the dominant C⋯H-D hydrogen bond. Nevertheless, also taking into account their large number, it is highly likely that their presence can influence the overall structure of the dimer. In particular, their presence can significantly influence the angle values as they are generally associated with small force constants.

#### 3.4.2. The IR${}_{2}\cdots$H${}_{2}$O and IR${}_{2}\cdots$MeOH Dimers

**Figure 7:**
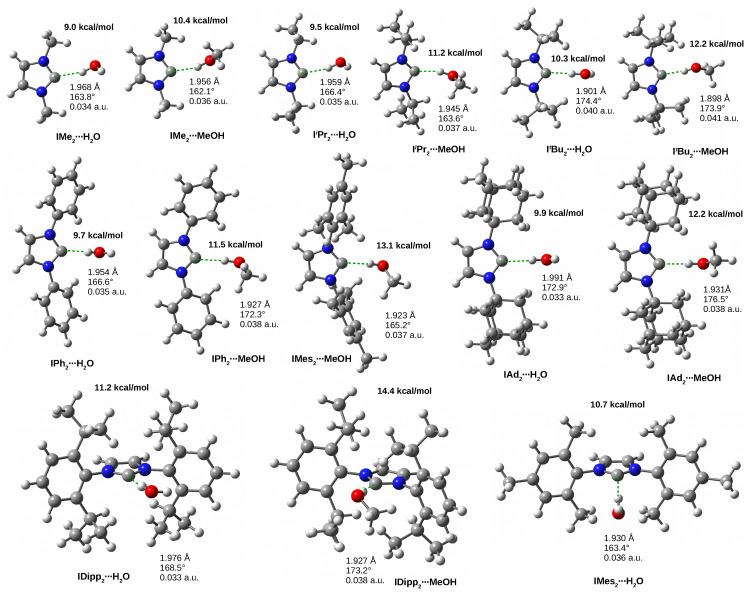
The IR${}_{2}\cdots$H${}_{2}$O and IR${}_{2}\cdots$MeOH (R = Me, ${}^{i}$Pr, ${}^{t}$Bu, Ph, Mes, Dipp, Ad) dimers. The values of the dissociation energy are given in bold, while the three values refer to the length of the hydrogen bond, the C-H-D angle and the value of the electron density at the bond critical point of the C⋯H-D hydrogen bond.

The significant non-linearity of the hydrogen bonds in the dimers I⋯H${}_{2}$O and I⋯MeOH already indicates the presence of additional intermolecular interactions, and indeed, as shown in Figure 4 and discussed previously, there is an additional hydrogen bond of the N-H⋯O type in both of these dimers. The structures of these dimers also prove that lone electron pairs on oxygen are readily available, and therefore, both H${}_{2}$O and MeOH will be willing to engage in the formation of accompanying hydrogen bonds. The resulting IR${}_{2}\cdots$H${}_{2}$O and IR${}_{2}\cdots$MeOH dimer structures are shown in Figure 7.

Among the dimers with water, IDipp${}_{2}\cdots$H${}_{2}$O and IMes${}_{2}\cdots$H${}_{2}$O are characterized by the highest dissociation energy value (11.2 and 10.7 kcal/mol, respectively), and the lowest is for IMe${}_{2}\cdots$H${}_{2}$O (9.0 kcal/mol). It is similar in the case of dimers with MeOH: IDipp${}_{2}\cdots$MeOH (14.4 kcal/mol), IMes${}_{2}\cdots$MeOH (13.1 kcal/mol), IMe${}_{2}\cdots$MeOH (10.4 kcal/mol). As was the case with dimers involving HF or HCN, the presence of any R substituent clearly increases the value of the dissociation energy. Clearly (Table 1, Figure 7), for a given carbene IR${}_{2}$, MeOH forms a stronger complex than H${}_{2}$O. This is also reflected in the shorter distances C⋯H. For example, for IPh${}_{2}$ the distances are 1.927 and 1.954 Å, respectively, and for IAd${}_{2}$, they are 1.931 and 1.991 Å, respectively. The dissociation energies for H${}_{2}$O are rather similar and generally slightly lower than those for HCN. It is worth noting that in the case of dimers with H${}_{2}$O and MeOH, the highest (i.e., most negative) values of $E_{b}$, i.e., the binding energy obtained by Equation (1), have been obtained for the I${}^{t}$Bu${}_{2}$ carbene, characterized by the formation of an almost linear (ca. 174${}^{\circ }$) C⋯H-O bond. On the contrary, the smallest values of $E_{b}$ have been obtained for IMe${}_{2}$ (−6.8 and −7.2 kcal/mol for H${}_{2}$O and MeOH, respectively). However, they are much larger than in the unsubstituted imidazol-2-ylidene (−5.3 and −5.5 kcal/mol, respectively).

**Figure 8:**
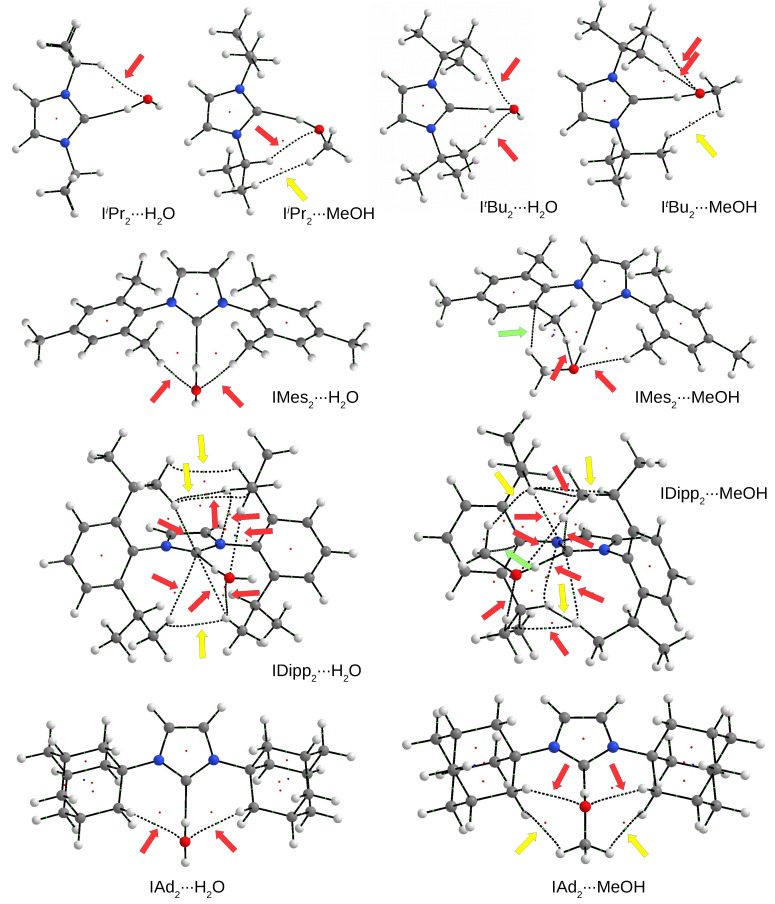
Molecular graphs of selected IR${}_{2}\cdots$H${}_{2}$O and IR${}_{2}\cdots$MeOH dimers. Arrows show the presence of bond paths for some accompanying interatomic interactions (hydrogen bonds—red, C-H⋯H-C contacts—yellow, and C-H⋯π hydrogen bonds—light green). Large balls represent atoms (hydrogen—white, carbon—gray, nitrogen—blue, and oxygen—red) and small balls represent critical points (bond critical points—light green, ring critical points—red, and cage critical points—blue).

Taking into account that the molecular graphs obtained for the IR${}_{2}\cdots$HF and IR${}_{2}\cdots$HCN dimers (Figure 6) in many cases indicated the presence of two, four or even more additional coexisting interactions, it is now worth analyzing the molecular graphs for IR${}_{2}\cdots$H${}_{2}$O and IR${}_{2}\cdots$MeOH dimers. The most interesting examples are shown in Figure 8.

In the dimers shown in Figure 8, generally one (e.g., I${}^{i}$Pr${}_{2}\cdots$H${}_{2}$O) or two (e.g., I${}^{t}$Bu${}_{2}\cdots$H${}_{2}$O) bond paths are present for the accompanying C-H⋯O hydrogen bonds (indicated by red arrows). It is clear, however, that for some carbenes, the MeOH dimers contain additional bond paths for C-H⋯H-C contacts (indicated by yellow arrows). Moreover, the presence of a methyl group in MeOH allows in some cases (see the dimers with IMes${}_{2}$ and IDipp${}_{2}$) an additional (i.e., compared with H${}_{2}$O) C-H⋯π-type hydrogen bond (green arrow). As was the case with the HF and HCN dimers, IDipp${}_{2}$ produces the most intricate molecular graph, suggesting the existence of many interactions accompanying the main C⋯H-O hydrogen bond involving the carbene carbon atom. In addition to three C-H⋯H-C interactions, numerous accompanying hydrogen bonds are present. Taking this into account, this fact may explain the exceptionally high value of dissociation energy in dimers with IDipp${}_{2}$, and especially in IDipp${}_{2}\cdots$MeOH (Table 1 and Figure 7). Nevertheless, all these accompanying interactions should be much weaker than the leading C⋯H-O hydrogen bond involving the carbene carbon atom. For example, C-H⋯O hydrogen bonds are generally in the range of −0.6 to −1.5 kcal/mol (e.g., −0.8 kcal/mol in I${}^{t}$Bu${}_{2}\cdots$H${}_{2}$O, −1.0 kcal/mol in I${}^{t}$Bu${}_{2}\cdots$MeOH, −1.1 kcal/mol in I${}^{i}$Pr${}_{2}\cdots$H${}_{2}$O and I${}^{i}$Pr${}_{2}\cdots$MeOH, and −1.4 kcal/mol in IMes${}_{2}\cdots$H${}_{2}$O), however they are slightly stronger in IAd${}_{2}\cdots$H${}_{2}$O (−1.6 kcal/mol) and especially in IAd${}_{2}\cdots$MeOH (−1.9 kcal/mol). The C-H⋯π bonds in IMes${}_{2}\cdots$MeOH and IDipp${}_{2}\cdots$MeOH are much weaker (−0.6 kcal/mol). On the other hand, the energy of C-H⋯H-C interactions is negligible, below −0.5 kcal/mol (e.g., −0.4 kcal/mol in IAd${}_{2}\cdots$MeOH). On the other hand, the *intra*molecular C-H⋯N hydrogen bonds in IDipp${}_{2}\cdots$H${}_{2}$O and IDipp${}_{2}\cdots$MeOH are significantly strong (−2.8 kcal/mol). All these energy values should be compared with the energies of the dominant C⋯H-O hydrogen bond, which in the case of IR${}_{2}\cdots$H${}_{2}$O and IR${}_{2}\cdots$MeOH dimers range from −6.6 to −8.4 kcal/mol (Table 1).

#### 3.4.3. The IR${}_{2}\cdots$NH${}_{3}$ Dimers

**Figure 9:**
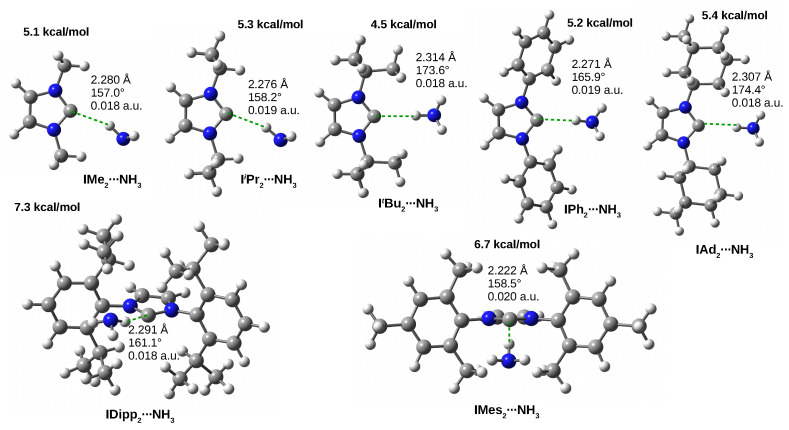
The IR${}_{2}\cdots$NH${}_{3}$ (R = Me, ${}^{i}$Pr, ${}^{t}$Bu, Ph, Mes, Dipp, and Ad) dimers. The values of the dissociation energy are given in bold, while the three values refer to the length of the hydrogen bond, the C-H-D angle and the value of the electron density at the bond critical point of the C⋯H-D hydrogen bond.

**Figure 10:**
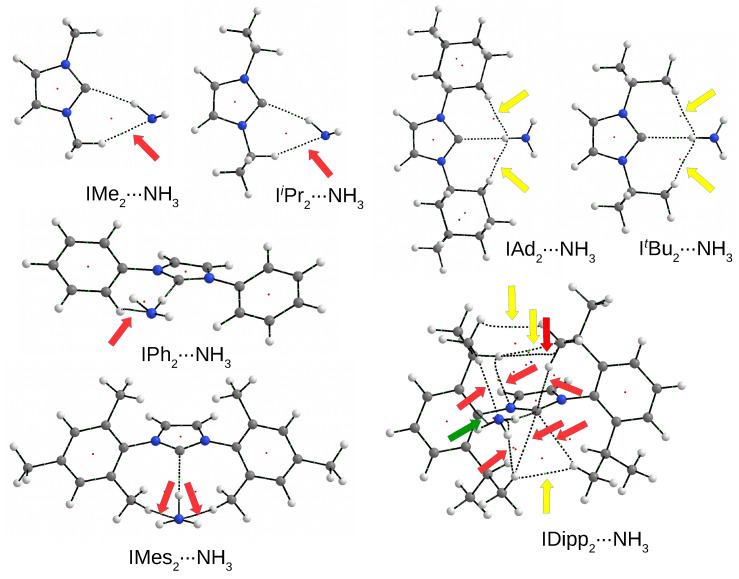
Molecular graphs of the IR${}_{2}\cdots$NH${}_{3}$ dimers. Arrows show the presence of bond paths for some accompanying interatomic interactions (hydrogen bonds—red, C-H⋯H-C or C-H⋯H-N contacts—yellow, and N⋯π contact—dark green). Large balls represent atoms (hydrogen—white, carbon—gray, and nitrogen—blue) and small balls represent critical points (bond critical points—light green, ring critical points—red, and cage critical points—blue).

The values of dissociation ($D_{0}$) and binding ($E_{b}$) energies listed in Table 1 show that the dimers with NH${}_{3}$ as well as the C⋯H-N hydrogen bonds in them should be the weakest. Namely, the $D_{0}$ values are from 4.5 kcal/mol in I${}^{t}$Bu${}_{2}\cdots$NH${}_{3}$ to 7.3 kcal/mol in IDipp${}_{2}\cdots$NH${}_{3}$. The $E_{b}$ values form a fairly narrow range from −3.2 kcal/mol (not including I⋯NH${}_{3}$ with a much lower value of −1.6 kcal/mol) to −3.7 for IDipp${}_{2}\cdots$NH${}_{3}$. Such small values of $D_{0}$ and $E_{b}$ may result from the fact that the N-H bond in ammonia is a worse proton donor than the O-H bond in water or methanol. The C-H-D angle ($\alpha_{\mathrm{CHD}}$) values show that in none of the dimers, the C⋯H-N hydrogen bond is linear (although in I${}^{t}$Bu${}_{2}\cdots$NH${}_{3}$ and IAd${}_{2}\cdots$NH${}_{3}$ this angle is ca. 174${}^{\circ }$); quite the opposite, the deviation from linearity is significant (157${}^{\circ }$–166${}^{\circ }$), although much smaller than in I⋯NH${}_{3}$ (123${}^{\circ }$). Such large deviations from linearity may indicate the presence of additional coexisting interactions. The structures of the IR${}_{2}\cdots$NH${}_{3}$ dimers are shown in Figure 9, while their molecular graphs can be found in Figure 10.

A careful comparison of the molecular graphs obtained for the IR${}_{2}\cdots$H${}_{2}$O and IR${}_{2}\cdots$MeOH dimers (Figure 8) with the molecular graphs obtained for the IR${}_{2}\cdots$NH${}_{3}$ dimers (Figure 10) shows some impoverishment in the second case. In addition, there is a visible change in the nature of some accompanying interactions in some systems with the same IR${}_{2}$ carbene. For example, the molecular graph of the I${}^{i}$Pr${}_{2}\cdots$NH${}_{3}$ dimer contains only one bond path for the interaction (C-H⋯N) accompanying the dominant C⋯H-N hydrogen bond, while I${}^{i}$Pr${}_{2}\cdots$MeOH in addition to the additional C-H⋯O also contains a bond path for C-H⋯H-C. The I${}^{t}$Bu${}_{2}\cdots$H${}_{2}$O dimer contains two bond paths for C-H⋯O hydrogen bonds, whereas I${}^{t}$Bu${}_{2}\cdots$NH${}_{3}$ also has two additional bond paths, but for C-H⋯H-C interactions. The situation is similar for the molecular graph involving Ad; in the case of dimer with H${}_{2}$O, there are two bond paths for C-H⋯O hydrogen bonds, whereas in the case of IAd${}_{2}\cdots$NH${}_{3}$, there are two bond paths for C-H⋯H-N. Keeping in mind that C-H⋯H-C interactions are weaker than C-H⋯O/N hydrogen bonds, together with a smaller number of accompanying interactions, this finding may explain the weaker strength of complexes with NH${}_{3}$. Similarly, it can be seen that IDipp${}_{2}$ creates many bond paths, of which as many as three (except three for N-H bonds, of course) lead to the nitrogen atom of the ammonia molecule. Two of them represent very weak C-H⋯N hydrogen bonds (−0.1 and −1.3 kcal/mol), while the other, interestingly, determines the N⋯π contact (−0.5 kcal/mol). Of course, all these interactions are much weaker than the dominant C⋯H-N hydrogen bond (−3.2 kcal/mol). Nevertheless, their multitude makes the dissociation energy for IDipp${}_{2}\cdots$NH${}_{3}$ clearly the highest (7.3 kcal/mol) among all the IR${}_{2}\cdots$NH${}_{3}$ dimers (see Table 1 or Figure 9). Of course, it should be noted that a similar dissociation energy value was obtained for a simple I⋯NH${}_{3}$ dimer (7.2 kcal/mol); however, as already shown (Figure 4), such a large value in this case results from the presence of a strong N-H⋯N hydrogen bond (−4.6 kcal/mol), which overwhelms the weaker C⋯H-N bond (−1.6 kcal/mol).

### 3.5. Relationship between Dimer Strength and the C⋯H Distance

**Figure 11:**
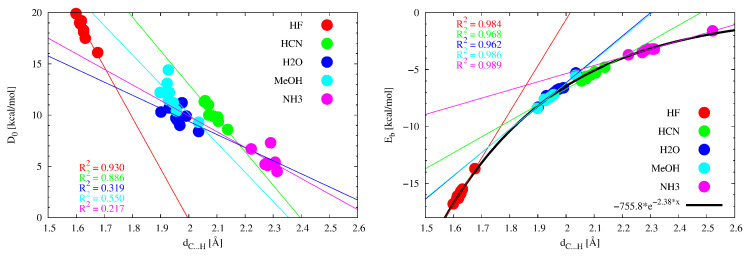
The dependence of either the dissociation energy (**left**) or the binding energy according to Equation (1) (**right**) on the distance C⋯H in the IR${}_{2}\cdots$HD dimers.

As has been shown in the previous subsections, many of the dimers considered here have some secondary interactions accompanying the leading C⋯H-D hydrogen bond. The strength of at least some of them may be considerable. Therefore, one should not expect a good overall (i.e., for all the IR${}_{2}\cdots$HD dimers) correlation between the dissociation energy, $D_{0}$, and the distance C⋯H. Moreover, good correlations are also not to be expected even in cases where individual proton-donor molecules are considered. Of course, the exception here is HF, and perhaps HCN, which more often forms linear or nearly linear C⋯H hydrogen bonds (Table 1). This is confirmed in Figure 11 (left), where it is shown that the $R^{2}$ (coefficient of determination) values for the linear correlation between $D_{0}$ and $d_{C\cdots H}$ are very poor for NH${}_{3}$ (even with the rejection of the clearly outlier point for I⋯NH${}_{3}$), H${}_{2}$O and MeOH (0.217, 0.319, 0.550, respectively) and much better for HCN (0.886) and especially for HF (0.930).

This result clearly shows that in the case of more complex dimers, i.e., having more significant intermolecular contacts, one should not expect a good relationship between the dimer dissociation energy and the length of any single intermolecular contact. Rather, a good correlation can be expected in the case of a clearly local parameter, which is the binding (interaction) energy determined from Equation (1), and therefore based on the electron density value at the critical point of the C⋯H-D hydrogen bond. Indeed, as shown in Figure 11 (right), for HF, MeOH and NH${}_{3}$, the linear correlations between $E_{b}$ and $d_{C\cdots H}$ are very good ($R^{2}$ > 0.98) and only slightly worse for HCN and H${}_{2}$O (0.968 and 0.962, respectively). Importantly, for all the IR${}_{2}\cdots$HD dimers, an excellent exponential relationship, $E_{b}=-755.8\;e^{-2.38\;d_{C\cdots H}}$, has been found (black curve in Figure 11). This result is not unexpected as a good common exponential relationship between $\rho_{\mathrm{bcp}}$ and the hydrogen bond length has been found for both different X and Y atoms in the X-H⋯Y hydrogen bond [128,129,130,131].

## 4. Conclusions

In this article, forty dimers formed by imidazol-2-ylidene or its derivatives, in which the hydrogen atoms of both N-H bonds were replaced by important and popular substituents of increasing complexity (methyl = Me, *iso*-propyl = ${}^{i}$Pr, *tert*-butyl = ${}^{t}$Bu, phenyl = Ph, mesityl = Mes, 2,6-diisopropylphenyl = Dipp, 1-adamantyl = Ad), and five fundamental proton donor (HD) molecules (HF, HCN, H${}_{2}$O, MeOH, NH${}_{3}$), have been studied. Although the most important goal was to describe the hydrogen bond formed by the carbene carbon atom, C⋯H-D, an equally important issue, which is, in the author’s opinion, insufficiently described in the literature, was the possibility of the formation of various accompanying interactions and their influence on the structure of the considered dimers. Despite different interaction abilities represented by carbenes and especially HD molecules, the following general trends have been found, in addition to many specific results:

- For a given carbene, dissociation energies of the IR${}_{2}\cdots$HD dimers increase in the following order: NH${}_{3}$< H${}_{2}$O < HCN ≤ MeOH ≪ HF.
- For a given HD molecule (HF, HCN, H${}_{2}$O, MeOH, or NH${}_{3}$), IDipp${}_{2}$, i.e., 1,3-bis[2,6-diisopropylphenyl]imidazol-2-ylidene, has been found to form the strongest dimers. This has been attributed to the multiplicity of various interactions accompanying the dominant C⋯H-D hydrogen bond.
- The substitution of hydrogen atoms in both N-H bonds of the imidazol-2-ylidene molecule by Me, ${}^{i}$Pr, ${}^{t}$Bu, Ph, Mes, Dipp or Ad groups leads to stronger dimers with HF, HCN, H${}_{2}$O or MeOH.

The article clearly shows that various types of secondary interactions, thus often omitted in the analysis, can have a significant impact on the structure of a molecular system and its strength. The results should improve our understanding of carbene chemistry and the role of intermolecular interactions.

## Figures and Tables

**Table 1:** Values of selected parameters characterizing the C⋯H-D hydrogen bond or the proton-donor molecule in the IR${}_{2}\cdots$HD dimers (I = imidazol-2-ylidene; R = Me, ${}^{i}$Pr, ${}^{t}$Bu, Ph, Mes, Dipp, Ad; HD = HF, HCN, H${}_{2}$O, MeOH, NH${}_{3}$): $D_{0}$–dissociation energy (in kcal/mol), $\rho_{C\cdots H}$–the electron density at the bond critical point of C⋯H-D (in a.u.), $E_{b}$–binding energy of the C⋯H-D hydrogen bond obtained by Equation (1) (in kcal/mol), $d_{C\cdots H}$–length of the C⋯H distance (in Å), $\alpha_{\mathrm{CHD}}$–the C-H-D angle (in degrees), $\Delta d_{\mathrm{HD}}$–elongation of the H-D proton-donor bond (in Å) relative to isolated HD (HF–0.917 Å, HCN–1.068 Å, H${}_{2}$O–0.958 Å, MeOH–0.957 Å, and NH${}_{3}$–1.013 Å).

HD	Property	Carbene							
		I	IMe ${}_{2}$	**I${}^{i}$Pr** ${}_{2}$	**I${}^{t}$Bu** ${}_{2}$	IPh ${}_{2}$	IMes ${}_{2}$	IDipp ${}_{2}$	IAd ${}_{2}$
HF	$D_{0}$	16.1	18.1	19.0	18.3	17.5	18.9	19.9	19.2
	$\rho_{C\cdots H}$	0.065	0.074	0.077	0.075	0.073	0.076	0.079	0.076
	$E_{b}$	−13.7	−15.7	−16.3	−15.9	−15.5	−16.1	−16.8	−16.3
	$d_{C\cdots H}$	1.676	1.626	1.612	1.626	1.632	1.614	1.599	1.618
	$\alpha_{\mathrm{CHD}}$	180.0	180.0	180.0	179.4	171.5	169.8	180.0	180.0
	$\Delta d_{\mathrm{HD}}$	0.061	0.075	0.080	0.084	0.075	0.080	0.083	0.088
HCN	$D_{0}$	8.6	9.4	9.9	10.0	9.8	11.0	11.4	11.3
	$\rho_{C\cdots H}$	0.025	0.027	0.028	0.029	0.027	0.029	0.030	0.030
	$E_{b}$	−4.8	−5.3	−5.5	−5.8	−5.2	−5.6	−5.9	−6.0
	$d_{C\cdots H}$	2.138	2.105	2.087	2.071	2.103	2.070	2.058	2.055
	$\alpha_{\mathrm{CHD}}$	180.0	180.0	180.0	180.0	173.7	167.2	179.7	180.0
	$\Delta d_{\mathrm{HD}}$	0.029	0.033	0.035	0.038	0.031	0.034	0.035	0.040
H${}_{2}$O	$D_{0}$	8.4	9.0	9.5	10.3	9.7	10.7	11.2	9.9
	$\rho_{C\cdots H}$	0.027	0.034	0.035	0.040	0.035	0.036	0.033	0.033
	$E_{b}$	−5.3	−6.8	−7.0	−8.3	−7.1	−7.3	−6.7	−6.6
	$d_{C\cdots H}$	2.035	1.968	1.959	1.901	1.954	1.930	1.976	1.991
	$\alpha_{\mathrm{CHD}}$	140.4	163.8	166.4	174.4	166.6	163.4	168.5	172.9
	$\Delta d_{\mathrm{HD}}$	0.019	0.025	0.026	0.033	0.026	0.028	0.023	0.027
MeOH	$D_{0}$	9.3	10.4	11.2	12.2	11.5	13.1	14.4	12.2
	$\rho_{C\cdots H}$	0.028	0.036	0.037	0.041	0.038	0.037	0.038	0.038
	$E_{b}$	−5.5	−7.2	−7.4	−8.4	−7.6	−7.6	−7.7	−7.6
	$d_{C\cdots H}$	2.035	1.956	1.945	1.898	1.927	1.923	1.927	1.931
	$\alpha_{\mathrm{CHD}}$	140.8	162.1	163.6	173.9	172.3	165.2	173.2	176.5
	$\Delta d_{\mathrm{HD}}$	0.018	0.025	0.026	0.032	0.026	0.026	0.026	0.030
NH${}_{3}$	$D_{0}$	7.2	5.1	5.3	4.5	5.2	6.7	7.3	5.4
	$\rho_{C\cdots H}$	0.011	0.018	0.019	0.018	0.019	0.020	0.018	0.018
	$E_{b}$	−1.6	−3.3	−3.4	−3.2	−3.5	−3.7	−3.2	−3.2
	$d_{C\cdots H}$	2.521	2.280	2.276	2.314	2.271	2.222	2.291	2.307
	$\alpha_{\mathrm{CHD}}$	123.1	157.0	158.2	173.6	165.9	158.5	161.1	174.4
	$\Delta d_{\mathrm{HD}}$	0.007	0.011	0.011	0.011	0.010	0.012	0.009	0.011

## Data Availability

Data available from the author on reasonable request.

## Abbreviations

The following abbreviations are used in this article:

NHC	N-heterocyclic carbene
I	imidazol-2-ylidene
IR${}_{2}$	R-substituted imidazol-2-ylidene
Me	methyl group
${}^{i}$Pr	isopropyl group
${}^{t}$Bu	*tert*-butyl group
Ph	phenyl group
Mes	mesityl group
Dipp	2,6-diisopropylphenyl group
Ad	adamantyl group
QTAIM	quantum theory of atoms in molecules
